# A descriptive and validation study of a predictive model of severity of SARS-COV-2 infection

**DOI:** 10.1515/almed-2021-0039

**Published:** 2021-05-27

**Authors:** Yolanda Villena-Ortiz, Marina Giralt, Laura Castellote-Bellés, Rosa M. Lopez-Martínez, Luisa Martinez-Sanchez, Alba Estela García-Fernández, Roser Ferrer-Costa, Francisco Rodríguez-Frias, Ernesto Casis

**Affiliations:** Department of Clinical Biochemistry, Laboratoris Clínics, Hospital Universitari Vall d’Hebron, Barcelona, Spain

**Keywords:** COVID-19, logistic model, SARS-CoV-2

## Abstract

**Objectives:**

The strain the SARS-COV-2 pandemic is putting on hospitals requires that predictive values are identified for a rapid triage and management of patients at a higher risk of developing severe COVID-19. We developed and validated a prognostic model of COVID-19 severity.

**Methods:**

A descriptive, comparative study of patients with positive vs. negative PCR-RT for SARS-COV-2 and of patients who developed moderate vs. severe COVID-19 was conducted. The model was built based on analytical and demographic data and comorbidities of patients seen in an Emergency Department with symptoms consistent with COVID-19. A logistic regression model was designed from data of the COVID-19-positive cohort.

**Results:**

The sample was composed of 410 COVID-positive patients (303 with moderate disease and 107 with severe disease) and 81 COVID-negative patients. The predictive variables identified included lactate dehydrogenase, C-reactive protein, total proteins, urea, and platelets. Internal calibration showed an area under the ROC curve (AUC) of 0.88 (CI 95%: 0.85–0.92), with a rate of correct classifications of 85.2% for a cut-off value of 0.5. External validation (100 patients) yielded an AUC of 0.79 (95% CI: 0.71–0.89), with a rate of correct classifications of 73%.

**Conclusions:**

The predictive model identifies patients at a higher risk of developing severe COVID-19 at Emergency Department, with a first blood test and common parameters used in a clinical laboratory. This model may be a valuable tool for clinical planning and decision-making.

## Introduction

In December 2019, China reported to the World Health Organization some cases of pneumonia of unknown etiology that has been detected in the region of Wuhan (Hubei, China) [1]. Later, the Chinese Center for Disease Control identified the causal agent as a β coronavirus, which was called SARS-CoV-2 causing COVID-19 disease [2]. Prior to the current pandemic, two β coronaviruses had been identified as the cause of two limited epidemic outbreaks: SARS-CoV-1 in 2003 and Middle East Respiratory Syndrome coronavirus (MERS-CoV) in 2012, with mortality rates near 10 and 36%, respectively [3]. Coronaviruses are a family of zoonotic viruses that may cause a range of diseases, from common cold to severe acute respiratory syndromes (SARS) that cause pulmonary infections and extrapulmonary manifestations [4].

Most SARS-CoV-2 patients remain asymptomatic or develop mild symptoms. However, 20% develop severe pulmonary disease characterized by fever, cough, dyspnea, pulmonary infiltrations, and acute respiratory syndrome, along with extrapulmonary manifestations [5]. In some patients, infection is associated with thromboembolism and an exacerbated immune response, resulting in a disproportionate release of pro-inflammatory cytokines, which has been described as a “cytokine storm” [6], [7]. These two situations, together with respiratory failure, has caused an increase in Intensive Care Unit (ICU) admissions and mortality [8].

SARS-CoV-2 infection rapidly developed into a pandemic of unpredictable effects and became a major public health problem [9]. Since the onset of the outbreak, efforts have focused on the search for and identification of clinical and analytical predictors of COVID-19 severity that guide immediate intervention in the most severe cases and the optimization of human and technical resources. The use of these predictors enables risk stratification in hospital units overwhelmed by unceasing admissions.

The clinical variables related to poor prognosis are advanced age, cardiovascular disease (CVD) or Chronic Obstructive Pulmonary Disease (COPD), diabetes mellitus (DM), arterial hypertension (HTN), dyslipidemia (DLP), and chronic kidney disease (CKD) [10], [11].

The clinical laboratory has played an essential role in the stratification of disease severity and prognosis. To such purpose, several analytes have been used, such as decreased oxygen pressure and saturation. Other useful analytes include lymphocyte count, elevation of inflammatory markers such as ferritin, C-reactive protein (CRP) and interleukin 6 (IL-6), increased prothrombin time (INR) and D dimer (DD), and elevation of enzymes such as lactate dehydrogenase (LDH), creatine kinase (CK), and aminotransferases, to name a few [12].

This situation has resulted in the proliferation of predictive models and artificial intelligence-based diagnostic, monitoring, and prognostic tools [13, 14].

The purposes of this study are: i) To conduct a descriptive study of a cohort of patients with symptoms consisted with SARS-CoV-2 infection seen in the Emergency Department (ED) of Hospital Universitari Vall d’Hebron; and ii) to develop and validate a predictive model based on an initial laboratory test that allows to identify SARS-CoV-2-positive patients at a higher risk of developing severe disease, ICU admission, and mortality.

## Materials and methods

### Study design and patient selection

A retrospective study carried out on April 23–30, 2020 in patients seen at the ED of Hospital Universitari Vall d’Hebron with symptoms consistent with SARS-CoV-2 infection. Blood and a nasopharyngeal and oropharyngeal swab were collected to test for SARS-CoV-2 at admission by real-time PCR (PCR-RT).

Based on PCR-RT result, patients were classified into two groups: COVID-positive and COVID-negative. All COVID-positive patients had been hospitalized and were classified into two subgroups: patients who developed moderate disease and patients with severe disease. Disease was considered to be severe if patients complied with the clinical criteria of severe disease at ICU admission and/or in case of death.

Exclusion criteria were: unavailability of clinical details; missing analyte or laboratory parameter (Table 1), and a positive PCR-RT result obtained during hospitalization of a patient previously classified as COVID-negative.

**Table 1:** Analytes and laboratory parameters included in the study and analytical method specifications.

Analytes and parameters	Analyzer	Specimen	Container
Interleukin 6 pg/mL	Cobas 411 (Roche)	Plasma	BD Vacutainer^®^ Barricor with lithium heparin

Hemoglobin, g/L	Sysmex XN-1000 (Roche)	Total blood	BD Vacutainer^®^EDTAK2
Hematocrit			
Red blood cell distribution width, %			
Leukocyte count, ×10^9^/L			
Neutrophil count, ×10^9^/L			
Lymphocyte count, ×10^9^/L			
Monocyte count, ×10^9^/L			
Platelet count, ×10^9^/L			

D-dimer, ng/mL	ACLTOP 750 (Werfen)	Plasma	BD Vacutainer^®^ with sodium citrate
Prothrombin time; INR			
Fibrinogen, g/L			

Alanine aminotransferase, IU/L	AU5800 (Beckman Coulter)	Plasma	BD Vacutainer^®^ Barricor with lithium heparin
Aspartate aminotransferase, IU/L			
Direct bilirubin, mg/dL			
Total bilirubin, mg/dL			
Calcium, mg/dL			
Creatinine, mg/dL			
Ferritin, ng/mL			
Glucose, mg/dL			
Lactate dehydrogenase, IU/L			
Potassium, mmol/L			
C-reactive protein, mg/dL			
Total protein, g/dL			
Sodium, mmol/L			
Urea, mg/dL			

SARS-CoV-2 coronavirus	Real-time polymerase chain reaction (RT-PCR). CFX96^™^ real-time system (BioRad)	Nasopharyngeal and oropharyngeal swab	Dedicated-vehicle container

Demographic data, medical history, and comorbidities were extracted from the hospital information system. The comorbidities considered were HTN, DLP, DM, CKD, COPD, and obesity.

This study was approved by the Ethics Committee of Hospital Universitari Vall d’Hebron.

### Laboratory analytes and parameters

The analytes studied and the analytical methods and specimen used are detailed in Table 1.

### Statistical analysis

#### Descriptive study

A descriptive study of the different groups was conducted to compare COVID-positive patients with COVID-negative patients, and moderate COVID-positive patients with severe COVID-positive patients. The variables included were age, sex, comorbidities, and the laboratory parameters detailed in Table 1.

Quantitative variables were expressed as median values and interquartile range (IR). Differences were assessed using Mann–Whitney U test for continuous variables, and Chi square test for dichotomous variables.

#### Predictive model

Multivariate logistic regression analysis to predict disease severity was developed based on data from the COVID-positive group (moderate and severe patients), in accordance with the guidelines of “Transparent Reporting of a multivariable prediction model for Individual Prognosis Or Diagnosis” (TRIPOD) [14]. The model initially included all the variables found to have predictive capacity on univariate analysis that are known to be predictive of severity and poor prognosis [15]. The maximum number of predictors that were included in the multivariate model was established in accordance with Peduzzi’s criteria [16] i.e. by the estimation of events per variable (EPV) as the number of events divided by the number of independent variables, with the coefficient being ≥10. A mixed and forward-backward stepwise variable selection procedure was employed.

Model-based diagnosis was established including an autocorrelation study (visual inspection of estimated residuals vs. predicted values); the absence of multicolinearity between predictors by variance inflation factor (VIF); control of outliers and leverage values, and ∆Beta [17], ∆*χ* ^2^, and ∆Dev [18]; interactions between predictors (by assessing all potential terms of interaction from an initial hierarchical maximum likelihood model and removing interactions if test result exceeded 0.05); and logit to assess linear relationships between variables (visual inspection by categorization of quantitative predictors based on *ntiles* by assigning the median to each category).

Model calibration was performed by the Hosmer & Lemeshow test. Predicted likelihoods were broken down into five groups, confirming goodness of fit if p>0.10. Application required that most of the expected frequencies were >5 and none was <1.

Internal validation of the model was performed using two approaches. First, cross-validation was performed based on a randomized categorization of data into k groups (n=5), estimating the model and calculating the goodness of fit of those groups. Second, we used the area under the curve (AUC) for *bootstrapping* [14].

Descriptive and regression analysis were carried out using Stata (version Stata/IC 15) software package.

#### External validation of the predictive model

External validation (EV) of the model was performed in an additional group of 100 COVID-positive patients admitted to the ED of Hospital Universitari Vall d’Hebron between June and October 2020 who were hospitalized and complied with inclusion criteria.

The resulting predictive model was applied to the validation group. The AUC was estimated and loss of prediction with respect to the original model was estimated (loss of prediction=AUC − AUC_VE_). The model was considered reliable if loss of prediction was <10%.

## Results

Of the 517 patients selected, 20 were excluded for meeting an exclusion criterion; 410 (82.5%) had a positive and 87 (17.5%) had a negative PCR-RT for SARS-CoV-2. Among positive patients, 303 (73.9%) developed moderate disease, whereas 107 (26.1%) developed severe disease. Of the latter, 61 patients (57.0%) died during the study.

Table 2 shows the demographic characteristics and associated comorbidities of patients, and a comparison of the group with moderate vs. the group with severe disease. The median age of COVID-positive patients was 61 years (IQR: 48–74), 55.4% were men, and the most frequent associated comorbidities were HTN (41.2%), DLP (27.3%), DM (18.8%) and heart disease (10.7%). The median age of patients with severe and moderate disease was 69 (55–78) and 60 years (48–73) (p<0.05), respectively. The group of patients with severe disease was primarily composed of men (66.4%). In addition, all associated comorbidities were more frequent in this group. Differences were statistically significant in CKD and heart disease (p<0.05).

**Table 2:** Demographic characteristics and comorbidities of SARS-CoV-2-positive patients; patients with moderate and severe disease.

		Total, n=407	Moderate, n=303	Severe, n=107	p-Value
**Age**		61.0 (48.0–74.0)	60.0 (48.0–73.0)	69.0 (55.0–78.0)	0.0001

**Sex**	Female	183 (44.6%)	147 (48.5%)	36 (33.6%)	0.008

**Comorbidities**	Arterial hypertension	169 (41.2%)	117 (38.6%)	52 (48.6%)	0.071
	Dyslipemia	112 (27.3%)	77 (25.4%)	35 (32.7%)	0.145
	Diabetes mellitus	77 (18.8%)	55 (18.2%)	22 (20.6%)	0.853
	Chronic kidney disease	20 (4.9%)	11 (3.6%)	9 (8.4%)	0.048
	Heart disease	44 (10.7%)	25 (8.3%)	19 (17.8%)	0.006
	Lung disease	32 (7.8%)	27 (8.9%)	5 (4.7%)	0.160
	Obesity	31 (7.6%)	20 (6.6%)	11 (10.3%)	0.216
	Other	19 (4.6%)	12 (4.0%)	7 (6.5%)	0.275

Results for dichotomous variables are shown as the number and percentage of patients. Differences between groups are expressed as the p-value of the Chi square test. Age is expressed as the median and interquartile range, and differences between groups are expressed as the p-value of Mann–Whitney U test.

Table 3 compares the values for the different analytes and laboratory parameters obtained for COVID-negative and COVID-positive patients. The same comparison is shown in Table 4 between patients with moderate vs. severe disease**.** In the group of COVID-positive patients, median values were significantly higher for fibrinogen, alanine aminotransferase (ALT), aspartate aminotransferase (AST), direct bilirubin, total bilirubin, ferritin, glucose, LDH, CRP, IL-6 and lower in red blood cell distribution width (RDW), leukocyte count, lymphocyte count, calcium, potassium, and sodium.

**Table 3:** Comparative study of analytes and laboratory parameters between COVID-positive and -negative patients.

Analytes and parameters	COVID negative patients, n=81	COVID positive patients, n=410	p-Value	Mann–Whitney
Interleukin 6 pg/mL	16.2 (7.8–36.9)	49.7 (27.0–83.8)	0.000	−6.771

Hemoglobin, g/L	12.8 (11.4–14.4)	13.6 (12.4–14.7)	0.004	−2.919
Hematocrit	40.4 (35.3–43.5)	41.5 (38.4–44.6)	0.015	−2.444
Red blood cell distribution width, %	13.2 (12.6–15.4)	13.1 (12.5–13.9)	0.048	1.98
Leukocyte count, ×10^9^/L	7.7 (6.0–9.9)	6.7 (5.2–8.8)	0.003	2.973
Neutrophil count, ×10^9^/L	5.2 (4.1–7.6)	5.0 (3.6–7.0)	0.251	1.148
Lymphocyte count, ×10^9^/L	1.4 (1.0–2.1)	1.0 (0.8–1.4)	0.000	4.91
Monocyte count, ×10^9^/L	0.6 (0.4–0.9)	0.4 (0.3–0.6)	0.000	6.05
Platelet count, ×10^9^/L	243 (185–307)	210 (165–262)	0.002	3.076

D-dimer, ng/mL	244 (149–582)	281.5 (183.0–543.5)	0.108	−1.607
Prothrombin time; INR	1.05 (0.99–1.12)	1.10 (1.03–1.16)	0.000	−3.652
Fibrinogen, g/L	4.7 (4.1–5.4)	5.65 (4.82–6.39)	0.000	−5.129

Alanine aminotransferase, IU/L	20 (15–28)	30.0 (19.0–49.8)	0.000	−4.216
Aspartate aminotransferase, IU/L	26 (20–33)	41 (30–57)	0.000	−7.208
Direct bilirubin, mg/dL	0.27 (0.22–0.33)	0.32 (0.25–0.39)	0.000	−3.923
Total bilirubin, mg/dL	0.54 (0.44–0.76)	0.62 (0.48–0.78)	0.075	−1.778
Calcium, mg/dL	9.2 (9.0–9.6)	9.0 (8.7–9.3)	0.000	4.209
Creatinine, mg/dL	0.80 (0.62–0.99)	0.82 (0.66–0.96)	0.281	−1.078
Ferritin, ng/mL	179 (94–519)	589.0 (300.5–1,088.3)	0.000	−6.272
Glucose, mg/dL	100 (89–113)	108 (96–130)	0.003	−3.007
Lactate dehydrogenase, IU/L	257 (217–303)	338.5 (281.3–437.0)	0.000	−7.168
Potassium, mmol/L	4.02 (3.67–4.23)	3.83 (3.60–4.05)	0.002	3.181
C-reactive protein, mg/dL	2.58 (0.67–8.91)	10.86 (5.05–18.16)	0.000	−6.695
Total proteins, g/dL	7.5 (7.1–7.9)	7.6 (7.1–8.0)	0.325	−0.985
Sodium, mmol/L	137.3 (135.4–138.5)	136.2 (134.2–138.2)	0.017	2.395
Urea, mg/dL	32 (24–50)	33.5 (26.0–46.8)	0.786	−0.271

Values are expressed as median and interquartile range. Comparative analysis is based on Mann–Whitney U test, which yielded p-value and percentages.

**Table 4:** Comparative study of analytes and laboratory parameters between patients with moderate and severe disease.

Analytes and parameters	Moderate, n=81	Severe, n=410	p-Value	Mann–Whitney
Interleukin 6 pg/mL	42.3 (21.1–65.6)	88.1 (61.2–133.0)	0.000	−8.787

Hemoglobin, g/L	13.6 (12.6–14.7)	13.4 (12.2–14.6)	0.246	1.161
Hematocrit	41.7 (38.7–44.6)	41.4 (38.2–44.2)	0.365	0.907
Red blood cell distribution width, %	13 (12.4–13.7)	13.4 (13.0–14.4)	0.000	−4.499
Leukocyte count, ×10^9^/L	6.5 (5.1–8.3)	7.4 (5.7–10.9)	0.000	−3.522
Neutrophil count, ×10^9^/L	4.8 (3.4–6.6)	6.1 (4.6–9)	0.000	−4.902
Lymphocyte count, ×10^9^/L	1.1 (0.8–1.5)	0.8 (0.6–1.1)	0.000	5.864
Monocyte count, ×10^9^/L	0.5 (0.3–0.6)	0.4 (0.3–0.5)	0.002	3.081
Platelet count, ×10^9^/L	216 (172–267)	195 (145–246)	0.012	2.521

D-dimer, ng/mL	258.0 (172.5–456.0)	424.0 (257.5–739.5)	0.000	−4.72
Prothrombin time; INR	1.1 (1.0–1.2)	1.1 (1.0–1.2)	0.719	−0.36
Fibrinogen, g/L	5.6 (4.8–6.3)	6.0 (5.0–6.6)	0.019	−2.34

Alanine aminotransferase, UI/L	29 (19–49)	31 (20–54)	0.3199	−0.995
Aspartate aminotransferase, IU/L	38 (29–52)	52 (36.5–71)	0.000	−4.991
Direct bilirubin, mg/dL	0.31 (0.25–0.38)	0.35 (0.27–0.45)	0.0005	−3.505
Total bilirubin, mg/dL	0.61 (0.48–0.75)	0.64 (0.48–0.86)	0.081	−1.743
Calcium, mg/dL	9.1 (8.8–9.3)	8.8 (8.5–9.0)	0.000	5.331
Creatinine, mg/dL	0.78 (0.64–0.92)	0.96 (0.83–1.30)	0.000	−6.886
Ferritin, ng/mL	481 (260–838)	986 (517–1,460)	0.000	−5.158
Glucose, mg/dL	105 (95–124)	120 (102–145)	0.000	−3.956
Lactate dehydrogenase, IU/L	314 (268–382)	450 (365–584)	0.000	−8.956
Potassium, mmol/L	3.80 (3.55–4.00)	3.96 (3.66–4.21)	0.001	−3.293
C-reactive protein, mg/dL	8.87 (3.83–15.30)	17.86 (11.19–23.57)	0.000	−7.491
Total proteins, g/dL	7.7 (7.3–8.0)	7.3 (6.9–7.7)	0.000	5.073
Sodium, mmol/L	136.2 (134.3–138.1)	136.2 (134.2–138.3)	0.687	−0.402
Urea, mg/dL	31.0 (24.0–41.0)	48.0 (33.0–72.8)	0.000	−7.294

Values are expressed as median and interquartile range. Comparative analysis is based on Mann–Whitney U test, which yielded p-value and percentages.

Patients with severe disease exhibited significantly higher values of IL-6, RDW, leukocyte count, DD, fibrinogen, AST, direct bilirubin, creatinine, ferritin, glucose, LDH, potassium, CRP, and urea, and lower values of lymphocyte and platelet count, calcium and total protein.

**Figure 1:**
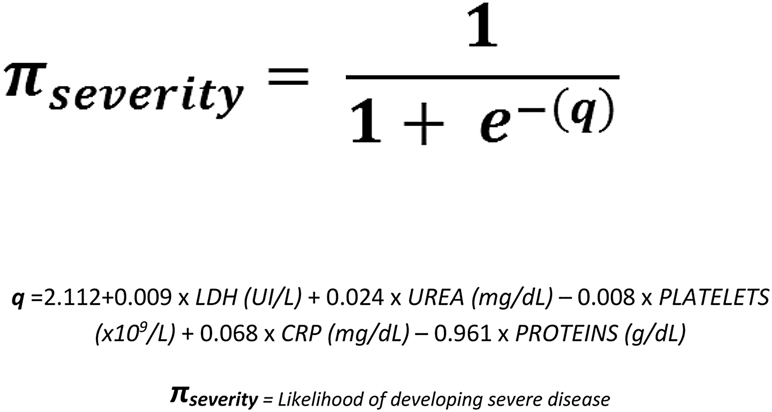
Logistic regression model function to predict COVID-19 severity.

The variables with the highest level of significance on multivariate analysis included: LDH, CRP, total proteins, urea and platelet count. The level of significance (Wald’s test) [19] for each variable was 6.43; 3.78; −3.78; 3.83, and −3.48, respectively. These variables complied with Peduzzi criterion, being 107 the number of events and 5 the predictors. Logistic model function [20] is displayed in Figure 1.

The predictive value of the model was 0.377. Global significance calculated by maximum likelihood estimation revealed that the model has a statistically significant predictive value for COVID-19 severity (χ^2^=173.55; df=5; p<0.05). As to goodness of fit, the following pseudo-R^2^ indices were obtained: Cox & Snell [21] of 0.353; Nagelkerke [22] of 0.515 and corrected Mcfadden [23] of 0.351.

**Figure 2:**
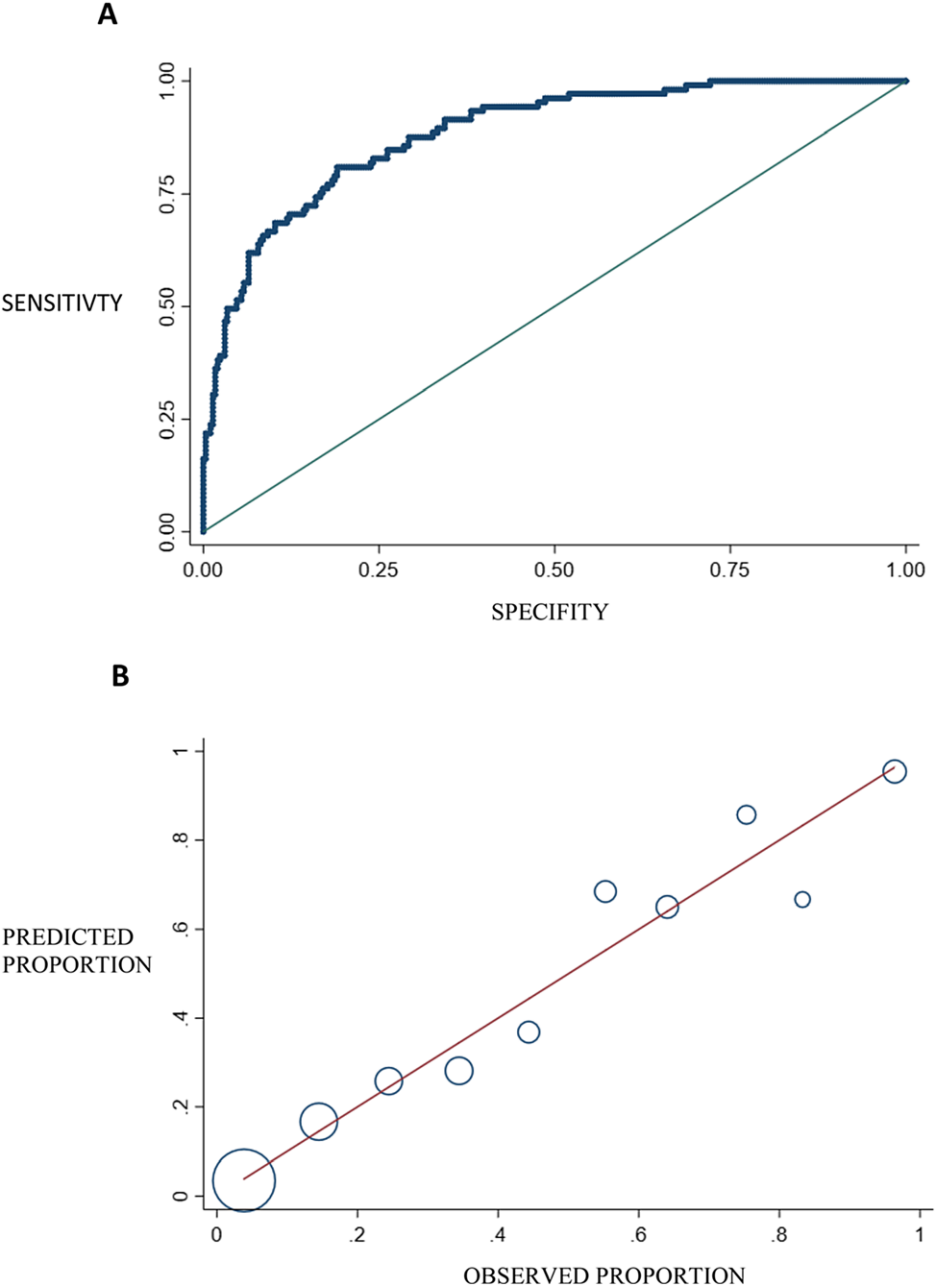
(A) Area under the ROC curve of the model to predict COVID-19 progression to severe disease. (B) Hosmer-Lemeshow test of the logistic regression model. The observed probability of severity is displayed on the X-axis, whereas predicted probability is displayed on the Y-axis. Bubble size is proportional to the number of patients.

AUC was 0.885 (95% CI: 0.849–0.921) (Figure 2A). For a cut-off of 0.5, diagnostic sensitivity (S) and specificity (Sp), positive predictive value (PPV) and negative predictive value (NPV) were 61.9, 93.5, 77.4, and 87.3%, respectively. The percentage of correct classifications was 85.2%. 60.0%, and 90.9%, respectively for a cut-off of 0.3 were 77.4, 81.6, 60.0, and 90.9%, respectively. In the latter, S increased as the percentage of correct classifications decreased.

In model diagnosis, independence of errors was confirmed visually (data not provided). The absence of multicolinearity between predictors was confirmed for all variables ( ${\bar{\text{x}}}_{\text{VIF}}=1.2$ ). Control of outliers and confounding factors led to the exclusion of two patients from the model (final_N_=408). Interaction between variables was ruled out, based on an initial hierarchical maximum likelihood model. Evaluation of linearity of variables based on logit was confirmed visually (data not provided).

Hosmer and Lemeshow test yielded a p=0.521, which exceeds the limit used in health sciences and confirms that the model was successfully calibrated (Figure 2B).

Internal validation was successful according to the two models employed: cross-validation yielded pseudo-R^2^ of 0.167–0.483; AUC estimation by *bootstrapping* was 0.840.

The sample used for external validation obtained an AUC of 0.94 (95% CI: 0.708–0.880), with a loss of prediction of 9.11%. S, Sp, PPV, and NPP for a cut-off of 0.3 were 73.7, 72.1, 77.8, and 67.4% respectively. The percentage of correctly classified patients was 73.0%. For a cut-off of 0.3, S, Sp, PPV, and NPP were 91.2, 51.2, 71.2, and 81.5%, respectively. S and NPV increased at the expense of Sp and PPV. The percentage of correctly classified patients was 74.0%.

## Discussion

This study provides data about demographics, comorbidities, and laboratory results of COVID-19 patients who were hospitalized during the first wave of the pandemic. It includes a comparative study of patients with moderate disease vs. patients who developed severe disease. We built a model for predicting COVID-19 progression to severe disease based on analytical parameters at first blood test performed at admission to the Emergency Department.

The results of this study confirm that the risk of disease severity is higher in males and increases with age. According to a cross-country mortality study where Spain took part [24], the man/women mortality ratio is 1.3. Hypotheses based on risk factors (alcohol use, smoking, lifestyle, regular medication, among others), and comorbidities, which vary with sex and age, could explain the differences observed in the study.

Patients with underlying comorbidities are at a higher risk of developing complications. The most frequent comorbidities in this study were HTN, DLP, DM, and heart diseases, followed by lung disease, obesity, and CKD. However, only heart disease and CKD were found to be more significantly associated with progression to severe disease and mortality [25]. The prevalence of all the comorbidities studied is widely documented in the literature [26]. It is worth mentioning that an underlying pro-inflammatory status and innate immunity diseases in patients with these chronic conditions could exacerbate symptoms, being associated with poor course and prognosis [27].

Alterations in different biological analytes (Tables 3 and 4) indicate multiorganic SARS-CoV-2 infection due to high expression of the cellular target of the virus, angiotensin-converting enzyme 2 (ACE2) [28].

The predictive model was built using the five analytes that better discriminated progression from moderate disease to severe disease i.e. elevated levels of LDH, CRP, and urea, and reduced levels of platelets and total proteins. During the study, the Service of Microbiology of our hospital only provided a qualitative result for the PCR-RT test for SARS-CoV-2. In addition, the relationship between disease severity and viral load of the sample is unclear.

LDH is a ubiquitous enzyme which increase indicates cell and tissue destruction. Increased LDH concentrations in plasma are considered a biomarker of activity and severity in pulmonary disease, being a prognostic marker of severe interstitial disease. In critical patients with COVID-19, elevated levels of LDH reflect increased disease activity and pulmonary damage [29] and are a marker of disease severity [30].

CRP is an acute-phase reactant that is significantly elevated at the early stages of infection by mediation of inflammatory factors such as IL-6. CRP has been used as a prognostic variable in acute respiratory distress syndrome [31]. CRP elevation is also suggestive of systemic vasculitis in patients with a poorer prognosis. The use of this marker for the prognosis of COVID-19 patients has been reported in several comparative studies involving patients with moderate vs. severe disease [32], [33] and has been associated with increased mortality [34].

Urea and creatinine are markers of kidney function. The two are filtrated in renal glomerulus, although creatinine is hardly reabsorbed in the tubules. Therefore, urea plays a relevant physiological role in glomerular tubular balance and is more sensitive than creatinine in the diagnosis of acute kidney failure (AKF). The increase in plasma urea has been associated with adverse effects and mortality in patients with heart failure [35], pulmonary thromboembolism [36], necrotizing pancreatitis [37], gastrointestinal bleeding [38], and pneumonia [39]. A recent study revealed that elevated levels of urea at admission are strongly associated with adverse events and mortality in patients admitted to the ICU, even after adjustment for kidney disease [40]. Increased urea in COVID-19 patients is an independent variable of poor prognosis [41], [42], probably associated with AKF, caused by hypoxemia resulting from respiratory distress or the direct action of the virus on renal tubules [43].

Reduced protein concentrations are interpreted as a surrogate marker of plasma albumin concentrations and have been associated with mortality in patients with pancreatitis, infection, trauma, burns, and liver dysfunction. The physiopathological mechanisms that mediate this reduction may result from an increase of vascular permeability and distribution volume, elevated expression of the vascular endothelial growth factor (VEGF), and a reduction of protein synthesis and shortening of the half-life of albumin. In acute infections, inflammation increases capillary permeability as a result of cytokine elevation and VEGF overexpression, leading to expansion of interstitial space and increasing the distribution volume of albumin [44]. A range of studies in patients with SARS-CoV-2 infection show that decreased plasma albumin concentrations is an independent predictive factor of adverse outcomes and mortality [45], [46]. However, these studies were unable to explain this condition in the first laboratory test in COVID-19 patients.

Thrombocytopenia has been documented to be associated with mortality in ICU patients [47]. COVID-19 patients with underlying inflammatory and systemic metabolic diseases frequently develop thrombocytopenia. The causes of thrombocytopenia are not well understood, although several hypotheses have been proposed: 1) platelet activation and aggregation due to direct pulmonary damage, resulting in the microthrombus formation and platelet consumption; 2) inhibition of platelet synthesis as a result of an insult to hematopoietic cells in the bone marrow secondary to inflammation and virus activity; 3) platelet destruction by the immune system [48]. A recent meta-analysis shows that thrombocytopenia is more prevalent in patients with severe COVID-19 and in patients who die from the disease [49]. It is still unclear whether thrombocytopenia is an independent risk factor of severity and death in these patients or is secondary to multiorganic failure. In our study, all patients with a poor prognosis at hospital admission showed thrombocytopenia. These results are consistent with a previous study demonstrating that a reduced platelet count in patients with severe disease anticipates the development of symptoms [50].

Although the model has a limited predictive power (37.7%), it has a high AUC (0.885), a high percentage of correct classifications (85.2%) and a PPV of 77.4% and a NPV of 87.3%, which reflect a good predictive value. The model was demonstrated to have been adequately calibrated, with predicted outcomes being consistent with real outcomes (Figure 2B).

The model was successfully tested for internal and external validation. However, in external validation, there was a loss of prediction of 9.1%. This may be explained by the small sample size used for validation or to substantial differences in the proportion of patients with severe disease in the original cohort (26.3%) vs. the validation cohort (57.0%).

Predictive models are very useful, since they provide key information to healthcare services and healthcare policy-makers. These models are based on underlying situations and data that may change as data are updated and reviewed. Therefore, there is some risk of bias if initial conditions are not satisfied. In this sense, it is important to be aware of the strengths and limitations of these models.

This model has some limitations. It is not applicable to patients with mild symptoms who do not require hospitalization, since the study was conducted in patients who were admitted to the ICU during the first wave of the pandemic. Another limitation is that concurrence of several comorbidities in each patient has not been assessed, which could affect the results of the study. Data about lifestyle habits such as alcohol abuse or smoking and other comorbidities including liver disease, immunodeficiency, and malignant neoplasms that could influence the course of the disease were not considered either. In addition, time from onset of symptoms to sample collection in the ED was not considered, since this information was not available in most patients. Analytes such as procalcitonin and troponin, associated with disease severity, were not considered in the laboratory test protocol for patients that entered at the ED of our Hospital and were not included. The predictive power of the model would probably improve with inclusion of these analytes. Further studies are required to investigate this possibility.

Despite its limitations, the model is a useful tool at the ED, since a simple routine blood test will help identify the patients at a higher risk of developing severe disease.
